# Supplementary material for: Personalised lifestyle recommendations for type 2 diabetes: Design and simulation of a recommender system on UK Biobank Data
Source: PLOS Digit Health. 2023 Aug 30;2(8):e0000333. doi: 10.1371/journal.pdig.0000333 (PMC10468058; doi:10.1371/journal.pdig.0000333)
Supplement: S1 Text — (DOCX) [file pdig.0000333.s001.docx]

# S1 Text. Normal ranges for biomarkers

- Age at onset > 45 years
- BMI < 30 kg/m2
- Waist circumference < 102 cm for males, 88 for females
- Glucose < 5.6 mmol/L
- HbA1c < 42 mmol/mol
- Systolic BP < 140 mmHg
- Diastolic BP < 90 mmHg
- Pulse rate 85-145 bpm
- Apolipoprotein A > 1.2 g/L
- Apolipoprotein B < 1 g/L
- Lipoprotein A < 75 nmol/L
- Triglycerides < 1.7 mmol/L
- Cholesterol < 5.2 mmol/L
- LDL direct < 2.6 mmol/L
- HLD-cholesterol > 1 mmol/L
- C-reactive protein < 2 mg/L
