# Supplementary material for: Personalised lifestyle recommendations for type 2 diabetes: Design and simulation of a recommender system on UK Biobank Data
Source: PLOS Digit Health. 2023 Aug 30;2(8):e0000333. doi: 10.1371/journal.pdig.0000333 (PMC10468058; doi:10.1371/journal.pdig.0000333)
Supplement: S2 Text — (DOCX) [file pdig.0000333.s002.docx]

# S2 Text. List of diseases within the CVD classification

Heart/cardiac problem, peripheral vascular disease, venous thromboembolic disease, angina, heart attack/myocardial infarction, heart failure/pulmonary oedema, heart arrhythmia, heart valve problem/heart murmur, cardiomyopathy, pericardial problem, stroke, transient ischaemic attack, subdural haemorrhage/haematoma, subarachnoid haemorrhage, leg claudication/ intermittent claudication, arterial embolism, deep venous thrombosis (DVT), pulmonary embolism +/- DVT, cerebral aneurysm, myocarditis, atrial fibrillation, rheumatic fever, atrial flutter, wolff parkinson white/WPW syndrome, irregular heartbeat, sick sinus syndrome, supraventricular tachycardia, brain haemorrhage, aortic aneurysm, other venous/lymphatic disease, varicose veins, lymphoedema, ischaemic stroke, mitral valve disease, aortic valve disease, hypertrophic cardiomyopathy, pericarditis, pericardial effusion, aortic aneurysm rupture, aortic dissection, varicose ulcer, mitral valve prolapses, mitral stenosis, mitral regurgitation/incompetence, aortic stenosis.
