# Supplementary material for: Personalised lifestyle recommendations for type 2 diabetes: Design and simulation of a recommender system on UK Biobank Data
Source: PLOS Digit Health. 2023 Aug 30;2(8):e0000333. doi: 10.1371/journal.pdig.0000333 (PMC10468058; doi:10.1371/journal.pdig.0000333)
Supplement: S3 Text — (DOCX) [file pdig.0000333.s003.docx]

# S3 Text. Compositional data and isometric log-ratio transformation

In a compositional framework, time spent in the three different activities is considered as a relative proportion of the overall time budget (24 hours), such that the vector $\mathbf{x}=\left[ x_{1},x_{2},\ldots,x_{D} \right]\in\mathbb{R}^{D}$, with $D=$ number of activities and with $\sum_{i}^{x} = C$, is constrained by the closure constant $C=24$ hours. The closure constant implies multicollinearity among the activities and thus conventional statistical methods cannot be employed with compositional data. The isometric log-ratio (ILR) transformation maps the data from the constrained simplex space to the unconstrained real space, which allows for the application of regression. Therefore, the transformation $z=ILR\left( x \right)$ is applied to the accelerometer data as follows:

$$z_{i}= \sqrt{\frac{D-1}{D-i+1}} \ln\frac{b_{i}}{\sqrt[D-i]{\prod_{j=i+1}^{d} b_{j}}} \mathrm{with}i=1,\ldots,D-1$$

In this paper we report only regression coefficients for $z_{1}$, since the regression coefficient $\beta_{1}$ for the first IRL coordinate $z_{1}$ represents the strength of the association between the chosen activity and the outcome, while $z_{1+i}$ cannot be interpreted in a meaningful way.

The ILR regression coefficients can be used to estimate the minutes of activity required to reduce the log(odds) of CVD, as Dumuid et al. proved that for a 3-activity composition the following holds:

\begin{equation}

$$\Delta CVD \left( k \right)= \beta_{1}\sqrt{\frac{3}{4} ln(k)}$$

$$k= \frac{1+r}{1-s}$$

$$s=r\frac{a_{i}}{a_{24h-i}}$$

with $k$ being change in daily activity composition, $r$ being the change in primary activity $a_{i}$ compared to the sample mean and $s$ being the corresponding change in the remaining activity components $a_{24h-i}$.
