# Supplementary material for: Personalised lifestyle recommendations for type 2 diabetes: Design and simulation of a recommender system on UK Biobank Data
Source: PLOS Digit Health. 2023 Aug 30;2(8):e0000333. doi: 10.1371/journal.pdig.0000333 (PMC10468058; doi:10.1371/journal.pdig.0000333)
Supplement: S4 Text — (DOCX) [file pdig.0000333.s004.docx]

# S4 Text. Ovid search strategy for review on barriers and facilitators to physical activity and sedentary behaviour

1. (physical train* or physical activ* or physical endur*).ti.
2. (sedentary behavio* or sedentary time* or sedentary lifestyle* or ((break* or interrupt*) adj3 sedentar* adj3 time*)).ti.
3. (barrier* or limitat* or imped* or restrict* or difficult*).ti.
4. (facilitat* or enable* or ease or assist*).ti.
5. ((type 2 diabetes or insulin-resistant diabetes or adult-onset diabetes or non-insulin dependent diabetes) not gestational not child*).ti.
6. 1 or 2
7. 3 or 4
8. 5 and 6 and 7
9. Remove duplicates from 8
10. ((behavio* change or intervention* or plan or program* or polic* or approach* or scheme or framework or guideline or concept) not trial).ti.
11. 9 and 10
12. Remove duplicates from 11
