# Supplementary material for: Personalised lifestyle recommendations for type 2 diabetes: Design and simulation of a recommender system on UK Biobank Data
Source: PLOS Digit Health. 2023 Aug 30;2(8):e0000333. doi: 10.1371/journal.pdig.0000333 (PMC10468058; doi:10.1371/journal.pdig.0000333)
Supplement: S1 Table — (DOCX) [file pdig.0000333.s005.docx]

# S1 Table. Sample sizes used in the analyses.

| **Analysis type** | **Description** | **n** |
| --- | --- | --- |
| Clustering | Participants with diabetes diagnosis and no missing data in the 16 biomarkers selected. | 14149 |
| Regression | Participants with diabetes, no missing data in the 16 biomarkers selected and accelerometer data. | 1805 |
| Regression sensitivity analysis | Participants with diabetes, no missing data in the 16 biomarkers selected, accelerometer data and no missing data in all confounders (age, waist circumference, income, smoking status). | 1615 |
| Survival analysis | Participants with diabetes, no missing data in the 16 biomarkers selected accelerometer data and older than 68 years. | 1215 |
| Classification into clusters | Participants with diabetes and older than 68 years (training set), participants with diabetes and younger than 68 years (test set). | 24600, 348 |
| Recommendation system simulation | Participants with diabetes, accelerometer data, younger than 68 years and with genetic data. | 290 |
| Recommender system effect at follow-up | Participants with diabetes, younger than 68 years, with genetic data and with accelerometer data measures both at baseline and follow-up. | 4 |
